# Effects of resuscitation with crystalloid fluids on cardiac function in patients with severe sepsis

**DOI:** 10.1186/1471-2334-8-50

**Published:** 2008-04-17

**Authors:** Zhi Xun Fang, Yu Feng Li, Xiao Qing Zhou, Zhen Zhang, Jin Song Zhang, Hai Ming Xia, Guo Ping Xing, Wei Ping Shu, Ling Shen, Guo Qing Yin

**Affiliations:** 1 The Second Hospital of Nanjing, affiliated with Medical School, Southeast University, 1-1 Zhong-fu Road, Nanjing, Jiangsu, 210003, P.R. China; 2 The First People's Hospital of Huai'an City, 3 Beijing Road, Huai'an City, Jiangsu, 223300, P.R. China; 3 The People's Hospital of Gaochun County, Gaochun County, Jiangsu, 211300, P.R China; 4 The Emergency Department of the First Hospital of Nanjing, affiliated with Nanjing Medical University, 68 Chang-le Road, Nanjing, Jiangsu, 210006, P.R. China; 5 The Emergency Department of the Jiangsu Province Hospital, affiliated with Nanjing Medical University. 300 Guangzhou Road, Nanjing, Jiangsu, 210029, P.R. China

## Abstract

**Background:**

The use of hypertonic crystalloid solutions, including sodium chloride and bicarbonate, for treating severe sepsis has been much debated in previous investigations. We have investigated the effects of three crystalloid solutions on fluid resuscitation in severe sepsis patients with hypotension.

**Methods:**

Ninety-four severe sepsis patients with hypotension were randomly assigned to three groups. The patients received the following injections within 15 min at initial treatment: Ns group (n = 32), 5 ml/kg normal saline; Hs group (n = 30), with 5 ml/kg 3.5% sodium chloride; and Sb group (n = 32), 5 ml/kg 5% sodium bicarbonate. Cardiac output (CO), systolic blood pressure, mean arterial pressure (MAP), body temperature, heart rate, respiratory rate and blood gases were measured.

**Results:**

There were no differences among the three groups in CO, MAP, heart rate or respiratory rate during the 120 min trial or the 8 hour follow-up, and no significant differences in observed mortality rate after 28 days. However, improvement of MAP and CO started earlier in the Sb group than in the Ns and Hs groups. Sodium bicarbonate increased the base excess but did not alter blood pH, lactic acid or [HCO_3_]^- ^values; and neither 3.5% hypertonic saline nor 5% sodium bicarbonate altered the Na^+^, K^+^, Ca^2+ ^or Cl^- ^levels.

**Conclusion:**

All three crystalloid solutions may be used for initial volume loading in severe sepsis, and sodium bicarbonate confers a limited benefit on humans with severe sepsis.

**Trial registration:**

ISRCTN36748319.

## Background

Severe sepsis has been recognized as an increasingly serious clinical problem, accounting for substantial morbidity and mortality. Critical status, organ hypoperfusion, volume deficiency and hypotension characterize its early phase. Therefore, initial care of patients with severe sepsis, such as early volume fluid loading and antibiotic treatment on admission, is emphasized as an early intervention [1-3]. In previous studies, the following benefits of crystalloid fluids have been demonstrated: (1) large-volume resuscitation with isotonic crystalloids conferred the highest survival rates; (2) extracellular fluid was redistributed during shock into both the intravascular and intracellular spaces; and (3) optimal resuscitation to correct this extracellular fluid deficit required infusion of a 3:1 ratio of isotonic crystalloid fluid and blood. Normal saline is the isotonic crystalloid fluid commonly used for volume loading in severe sepsis [2-4].

Small volume resuscitation with hypertonic saline appears to be effective for patients with sepsis or models of experimental severe sepsis [1]. In previous studies, hypertonic saline in dextran improved the hemodynamics of sepsis patients more effectively than an equivalent volume of normal saline [5-7]. Experimental studies in severe sepsis have also shown beneficial effects [8-11]. In rabbits with septic shock, 5 ml/kg hypertonic (3.5%) saline without colloid induced a decline in cardiac contractility and a decrease in mean arterial pressure (MAP), and eventually resulted in the death of the animals [12]; whereas in macaque models, the same fluid resuscitation improved myocardial performance [13,14]. We believe that hypertonic saline differs in effectiveness between primates and non-primates. Therefore, hypertonic crystalloid resuscitation of severe sepsis patients needs to be investigated in clinical trials.

Sodium bicarbonate has been used clinically for resuscitating sepsis patients, but its efficiency against acidosis and septic shock is the subject of widespread controversy [13-28]. In clinical practice before the 1990s, perfusion with 5% sodium bicarbonate at 5 ml/kg was recommended for fluid resuscitation of severe sepsis and septic shock patients [15-19]. The reasons were as follows. (1) Hypoperfusion in a single organ resulted in hypoxia and lactic acidosis in this organ, but the arterial blood pH might be normal in early septic shock and severe sepsis. Therefore, normal pH in septic shock did not contraindicate bicarbonate. (2) Acidosis impairs myocardial performance and alters renal blood flow; alkalinized blood might improve cardiovascular performance [20,21]. (3) Hypertonic sodium bicarbonate appeared to be effective for volume replacement. However, it has recently been shown that acidosis might have a protective effect. A low pH has been shown to delay the onset of cell death, but accelerating pH correction removes the protective effect and accelerates cell death. Also, acidosis during reperfusion limits the size of myocardial infarctions [22-25]. In experimental investigations of septic shock, sodium bicarbonate aggravated the pathophysiological state in non-primates such as rats [12], rabbits [26] and ponies [27]. There is no agreement about the use of sodium bicarbonate for treating severe sepsis, and it has not been commonly recommended in clinical practice since the 1990s [28]. However, the effect of sodium bicarbonate in primates is different. We recently demonstrated that administration of sodium bicarbonate improved the performance of the myocardium and normalized the hemodynamic parameters in macaque models [13,14]. The macaque model data suggest that 5% sodium bicarbonate is beneficial in fluid resuscitation during early phase septic shock in primates. We believe that the effects of sodium bicarbonate differ between primates and non-primates because of the physiological difference between the two types of animals. In view of our findings in macaques, we consider that sodium bicarbonate might be effective for treating severe sepsis and early septic shock in humans because macaques and humans are both primates and are physiologically similar.

The above findings enable us to speculate that crystalloid solutions, whether isotonic normal saline or hypertonic crystalloid fluids, are effective for initial volume loading in severe sepsis, and to consider that the effect of crystalloid solutions on severe sepsis should be assessed. Five percent sodium bicarbonate, with an osmotic pressure of 1190 mOsm/l, is hypertonic. The osmolality of 3.5% sodium chloride (1197 mOsm/l) is similar to that of 5% sodium bicarbonate, and this solution was used to control osmolality in the rabbit and macaque studies [12,13]. Therefore, a contrast-randomized clinical protocol of equal volumes (5 ml/kg) of various solutions, namely normal saline, 5% sodium bicarbonate and 3.5% sodium chloride, was designed in the present study.

Over a period of about twenty years, many authors have investigated the physiological signs of septic shock and their treatment [29-34]. Initial treatment of septic patients is critical for ensuring their survival, and therapy for severe sepsis is emphasized as an early intervention. Crystalloid solutions are effective as immediate perfusions for initial volume replacement, but are effective for only 2–3 hours [34]. Our study, therefore, focused on the initial effects of three crystalloid solutions on early-phase severe sepsis, 120 min after the administration of the bolus of fluid.

## Methods

This is a prospective, randomized and multicentric study conducted in five hospitals of Jiangsu Province, China: the Second Hospital of Nanjing, affiliated with Medical School of Southeast University; the First People's Hospital of Huai'an City; the People's Hospital of Gaochun County; the Emergency Department of the First Hospital of Nanjing, affiliated with Nanjing Medical University; and the Emergency Department of the Jiangsu Province Hospital, affiliated with Nanjing Medical University. This research was carried out in accordance with the Declaration of Helsinki (2000) of the World Medical Association. The protocol was approved by the Health Office of Jiangsu Provincial Government and by all the hospital ethics committees. Five hundred and thirty-two patients with severe sepsis in these five hospitals were diagnosed between Jun 01, 2001 and Oct 31, 2005, and 94 of the 532 subjects were enrolled in the study.

### Enrolment

Severe sepsis and septic shock were defined according to the criteria reported by Bone and colleagues. Hypotension is determined by a systolic blood pressure lower than 90 mm Hg or a reduction of 40 mm Hg from baseline. SIRS criteria consist of a suspected sepsis source and two of the four SIRS criteria (temperature >38°C or <36°C, heart rate >90 beats/min, respiratory rate >20 breaths/min, PaCO_2 _<32 mm Hg, or white blood cell count >12,000 cells per mm^3^, <4,000 cells per mm^3^, or >10% band cells). The status of shock patients may include but is not limited to lactic acidosis, oliguria or an acute alteration in mental state [35].

The criteria for patient enrolment were: (1) hypotension, namely, systolic blood pressure lower than 90 mmHg, or MAP lower than 70 mmHg, or a reduction of 40 mm Hg from baseline; (2) two of four SIRS, or a positive blood culture. Items (1) and (2) were necessary for enrolment.

All the enrolled patients signed informed consent documents within 1 h of arrival, and child patients had their informed consent documents signed by their guardians.

### Exclusion

Patients with any of the following were excluded: myocardial infarction, hemorrhagic shock, trauma, pregnancy, do-not-attempt-resuscitation orders, requirement for immediate surgery, or death imminent within 24 hours.

In order to focus the study on early phase severe sepsis, patients with final-phase septic shock characterized on admission by coma, seizure, diffuse intravascular coagulation (DIC), pulmonary edema and anuria were rejected. To determine the effect of crystalloid solutions on hemodynamics, cases treated with vasopressors, inotropic agents, colloids and mechanical ventilation during the initial two hours after admission were excluded because those treatments may markedly influence cardiovascular function.

### Scores for physiology of patients

The Acute Physiology and Chronic Health Evaluation (APACHE) II score, Simplified Acute Physiology Score (SAPS) II and Sequential Organ Failure Assessment Score (SOFA) were computed for all the patients from physiological measurements obtained on admission. Expected mortality rates for APACHE II and SAPS II scores were calculated using the logistic regression calculations suggested by Knaus and Le Gall [36-38]. The clinical data are summarized in Table 1.

**Table 1:** Clinical features, baseline demographic and physiological variables on admission in the three groups

	Ns group	Hs group	Sb group
Age (means ± SD)	44.19 ± 22.51	49.17 ± 21.39	32.59 ± 19.08^†^
median (min-max)	39(5–79)	50(5–83)	31(15–76)
Male/Female	20/12	19/11	15/17
APACHEA II score	18.69 ± 3.69	19.97 ± 4.23	18.00 ± 3.82
Predictive mortality by APACHE II	42.94% ± 13.30%	49.13% ± 18.22%	40.38% ± 15.48%
SAPS II score	29.31 ± 8.05	33.59 ± 9.76	27.03 ± 9.52
Predictive mortality rate for SAPS II	12.16% ± 9.58%	17.80% ± 12.54%	10.69% ± 10.59%
SOFA	8.97 ± 1.82	9.17 ± 2.46	8.44 ± 2.00
Observed mortality	5/32(15.63%)	5/30(16.67%)	5/32 (15.63%)
**Diagnosis**			
Acute enteritis	1		1
Acute nonlymphoblastic leukemia	11	13	7
Agranulocytosis		1	
Aplastic anemia		1	2
Bacillary dysentery	5	3	3
Bronchopneumonia	1		
Cholecystitis	2	3	2
Chronic myeloid leukemia	3	1	
Fulminent N meningitis		1	
Kidney cancer		1	1
Multiple trauma	2	1	2
Peritonitis			2
Pneumonia	3	2	7
Osteomyelitis	1	1	
Septicemia	2	3	6
Urinary tract infection	1		1
**Microbiology**			
Acinetobacter baumannii	1		
Candida albicans	4	2	2
Escherichia aerogenes	1		
Escherichia cloacae	1	4	
Escherichia coli	5	9	9
Gram-negative bacilli	4	1	2
Gram-positive bacilli	2	2	6
Pseudomonas aeruginosa		2	1
Mucor		2	
Myososis		1	
N. meningococcus		1	
Shigella boydii	3	1	1
Shigella dysenteriae	3	2	3
Staphylococcus	1		3
Streptococcus viridans	1	1	
Negative	6	2	5

Values are expressed as mean ± standard deviation. Compared with the Ns group, ^† ^p < 0.05. APACHE, Acute Physiology and Chronic Health Evaluation score; SAPS, Simplified Acute Physiology Score; SOFA, Sequential Organ Assessment.

### Program of treatment

Using a randomized block design, the 94 patients were randomly assigned to three groups. Patients in these groups received the following injections within 15 min of initial treatment: Ns group (n = 32), 5 ml/kg normal saline; Hs group (n = 30), 5 ml/kg 3.5% sodium chloride; and Sb group (n = 32), 5 ml/kg 5% sodium bicarbonate. At 100 min after T_0, _all patients were injected with 20 ml/kg 0.9% normal saline. None of the patients received any additional therapy such as vasopressors, colloids or mechanical ventilation within 120 min of the trial.

At T_120 _(120 min after T_0_), all patients were treated according to management guidelines for severe sepsis and septic shock [39]. The therapy that best suited the physiological status of the patient was performed, including administration of vasopressors/inotropics, colloid resuscitation, blood product transfusion or mechanical ventilation. Broad spectrum antibiotics were given to all patients and were altered according to blood culture and sensitivity findings.

### Outcome measures

Cardiac output (CO), systolic blood pressure, mean arterial pressure (MAP), body temperature, heart rate and respiratory rate were measured at the time that fluid resuscitation was started (T_0_); measurements were taken once every 30 min for 120 min after T_0 _(T_30_, T_60_, T_90 _and T_120_) and once at the 8 h point after T_0 _(T_8h_). Blood gases were measured at T_0 _and T_120_. CO was detected using color Doppler echocardiography [Aloka-SSD 5000-SV, Japan, or LOGIQ BOOK portable ultrasound scanners, GE, USA], and the CO parameters were calculated according to the recommendations of the American Society of Echocardiography [40,41].

Random grouping of cases and registration of parameters were operated by professional statisticians at the hospitals rather than by physicians. CO was measured by a professional echocardiographic doctor.

### Statistical analysis

Data were analyzed using SPSS for Windows, v13.0 (SPSS Inc, Chicago, IL, USA). A two-way ANOVA, with one of the ways being repeated measures, was performed first. Then a statistician tested for differences between the groups directly and for differences within a group. All physiological values are given as means ± SD, but age is presented as mean ± SD and median (minimum-maximum). Significance was defined as *p *< 0.05.

## Results

### Patient outcomes

The demographic data are presented in Table 1. Before solution resuscitation was started there were no significant differences among the three groups in severity scores (APACHE II, SAPSII and SOFA). When the expected mortalities in the Ns group were compared with those in the Hs or the Sb group by the APACHE II and SAPS II scores, no significant differences were found (Table 1). Intervention was completed in all 94 patients in 120 min, and the follow-up was completed in 8 h. Five patients in the Ns group died on the 6^th^, 10^th^, 18^th^, 20^th ^and 25^th ^days following intervention; 5 in the Hs group died on the 7^th^, 9^th^, 15^th^, 20^th ^and 25^th ^days; and 5 in the Sb group died on the 6^th^, 10^th^, 15^th^, 19^th ^and 25^th ^days. There were no significant differences in mortality rates between the Ns, Hs and Sb groups.

### Cardiac output and blood pressure

On admission, all the patients had abnormal temperature, abnormal white blood cell count, hyperpnea, tachycardia, decreased CO and hypotension.

**Figure 1:**
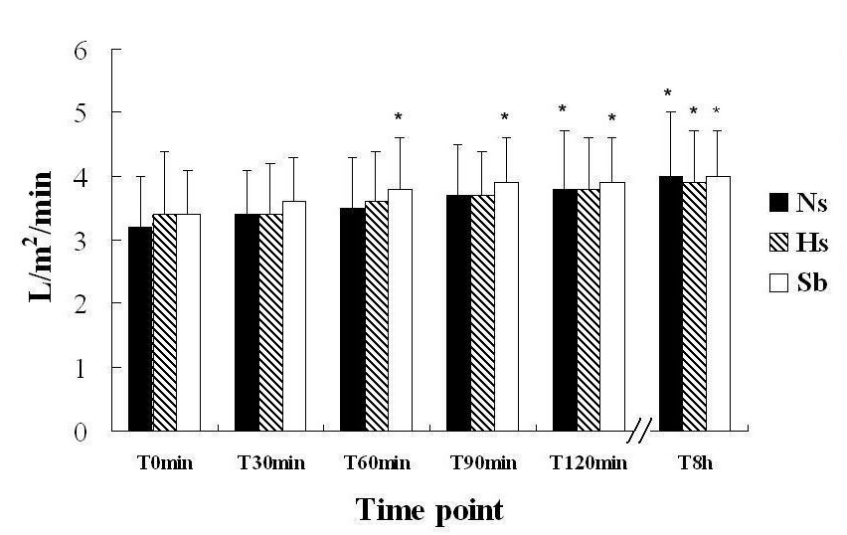
**Effects of fluid resuscitation on CO in the three groups**. The trial program and case grouping were carried out according to the design indicated in Methods. CO was measured by Doppler echocardiography. There were no differences in CO among the three groups at all the six time points. Comparing the CO variables in the same group, CO at T_120 _and T_8h _was significantly higher than at T_0 _in the Ns group; the parameter at T_8h _was higher than at T_0 _in the Hs group; and CO at T_60_, T_90_, T_120 _and T_8h _were higher than that at T_0 _in the Sb group (*p < 0.05). CO improved earlier in the Sb group than in the Ns and Hs groups.

Echocardiograms were recorded by the Teichholz method from the left parasternal window, and the parameters of cardiac function such as CO were read directly. At T_0_, the CO was 3.17 ± 0.79 l/min in the Ns group, 3.37 ± 0.99 l/min in the Hs group and 3.34 ± 0.65 l/min in the Sb group. CO was compared at serial time points after fluid resuscitation with that at T_0 _in the same group: it increased significantly at T_120 _in the Ns group, at T_8h _in the Hs group and at T_60 _in the Sb group (Fig. 1). CO improved earlier in the Sb group than in the Ns or Hs groups. However, there were no differences in CO among the three groups at the same time points.

**Figure 2:**
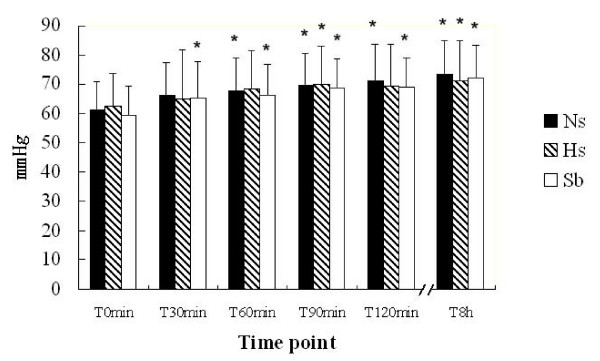
**Effects of fluid resuscitation on MAP in the three groups**. MAP in all patients was registered by professional statisticians in the hospitals. There were no differences in MAP among the three groups at all the six time points. Comparing MAP in the same group, MAP at T_60_, T_90_, T_120 _and T_8h _was significantly higher than that at T_0 _in the Ns group; the parameter at T_90_, T_120 _and T_8h _was higher than at T_0 _in the Hs group; and MAP at T_30_, T_60_, T_90_, T_120 _and T_8h _were higher than that at T_0 _in the Sb group (*p < 0.05). MAP increased earlier in the Sb group than in the Ns and Hs groups.

In all three groups, MAP at T_0 _was lower than 70 mm Hg: 61.21 ± 9.69 mm Hg in the Ns group, 62.55 ± 10.95 mm Hg in the Hs group and 59.38 ± 9.79 mm Hg in the Sb group. Normal saline treatment resulted in a marked increase of MAP at T_60_, 3.5% sodium chloride at T_8h _and 5% sodium bicarbonate at T_30_. MAP improved earlier in the Sb group than in the Ns and Hs groups. However, there were no differences in MAP among the three groups at the same time points (Fig. 2).

**Figure 3:**
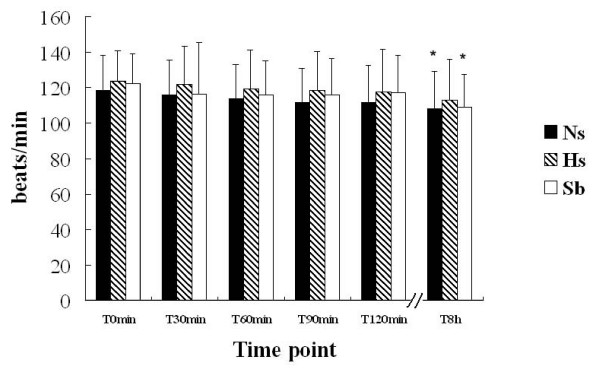
**Effects of fluid resuscitation on heart rate in the three groups**. Heart rate in all cases was recorded by statisticians. There were no differences in heart rate among the groups during the 120 min trial or the 8 h follow-up. Comparing heart rate in the same group, the heart rate at T_8h _was lower than that at T_0 _in the Ns and Sb groups (*p < 0.05), but no change of heart rate appeared in the Hs group. Resuscitation by fluids did not change heart rate in patients during the 120 min trial.

**Figure 4:**
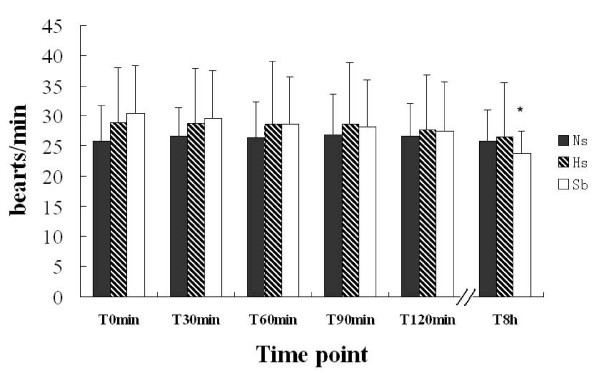
**Effects of fluid resuscitation on respiratory rate in the three groups**. There were no differences in respiratory rate among the groups during the 120 min trial or the 8 h follow-up. Comparing respiratory rates in the same group, the respiratory rate at T8h was lower than that at T0 in the Sb group (*p < 0.05) only; no change of respiratory rate appeared in the Ns or Hs groups. Resuscitation by fluids did not change the respiratory rate of patients during the 120 min trial.

On admission, the patients in all three groups suffered from hyperpnea and tachycardia. Fluid resuscitation did not alter the heart rates or respiratory rates after 120 min in any of the three groups, but caused a decrease of heart and respiratory rates in the Sb group and a decrease of heart rate in the Ns group at T_8h _(Figs. 3 and 4).

### Acid-basic balance and electrolytes

The blood pH, lactic acid and [HCO_3_]^- ^values in the three groups were not altered during the 120 min following treatment. The base excess (BE) values in the Ns and Hs groups were not changed at T_120 _whereas those of the Sb group were increased. The BE values of the Sb group at T_120 _were higher than those of the Ns group. Sodium bicarbonate improved the BE values (Table 2).

**Table 2:** Changes of acid-base balance and electrolytes over 120 min in the three groups

	Ns group	Hs group	Sb group
pH			
T_0_	7.33 ± 0.16	7.40 ± 0.11	7.38 ± 0.12
T_120_	7.34 ± 0.12	7.36 ± 0.16	7.44 ± 0.06
BE-B			
T_0_	-7.21 ± 6.27	-5.26 ± 6.57	-4.10 ± 7.06
T_120_	-5.62 ± 6.83	-5.83 ± 8.76	1.64 ± 4.23* ^†^
[HCO_3_]^- ^(mmol/l)			
T_0_	18.43 ± 4.87	17.76 ± 6.51	20.84 ± 5.74
T_120_	19.77 ± 6.94	16.43 ± 7.84	24.27 ± 6.08
Lactic acid (mmol/l)			
T_0_	8.15 ± 8.05	3.35 ± 0.21	4.30 ± 1.98
T_120_	1.37 ± 0.11	3.15 ± 0.07	3.48 ± 1.70
Na^+ ^(mmol/l)			
T_0_	135.91 ± 7.21	135.86 ± 9.52	135.15 ± 7.00
T_120_	136.70 ± 5.90	135.62 ± 10.19	138.57 ± 6.89
K^+ ^(mmol/l)			
T_0 min_	3.93 ± 0.84	3.78 ± 0.71	4.02 ± 0.47
T_120 min_	4.11 ± 0.71	3.83 ± 0.56	3.77 ± 0.59 ^†^
Ca^2+ ^(mmol/L)			
T_0_	2.12 ± 0.28	1.96 ± 0.50	2.35 ± 0.43
T_120_	2.17 ± 0.33	2.05 ± 0.36	2.28 ± 0.53
Cl^- ^(mmol/l)			
T_0_	94.49 ± 18.58	97.90 ± 10.89	99.02 ± 4.40
T_120_	98.41 ± 5.18	97.78 ± 5.10	98.93 ± 3.39
BUN (mmol/l)			
T_0_	9.75 ± 8.48	7.53 ± 4.85	7.99 ± 7.33
T_120_	9.00 ± 8.16	10.09 ± 6.25	7.74 ± 7.96

Values are expressed as mean ± standard deviation. Compared with T_0 min _in the same group, * *p *< 0.05. Compared with Ns group at the same time point, ^† ^*p *< 0.05.

Fluid resuscitation, whether with normal saline, 3.5% sodium chloride or 5% sodium bicarbonate, did not alter the Na^+^, K^+^, Ca^2+ ^or Cl^- ^levels in any of the three groups. However, K^+ ^in the Sb group was lower than that in the Ns group at T_120_. Blood urea nitrogen was not altered in any group.

## Discussion

The major findings of the present study were: that in patients with severe sepsis, there were no differences in CO, MAP, heart rate or respiratory rate between the three groups during the 120 min trial or the 8 h follow-up; that there were no significant differences between the Ns group and the Hs or Sb groups in observed mortality rate at 28 days; and that sodium bicarbonate improved MAP and CO more rapidly than normal saline or hypertonic sodium chloride. However, the benefits of sodium bicarbonate were limited.

Previous studies have demonstrated that initial crystalloid volume loading is valuable in achieving hemodynamic stability [1-4]. Consistent with previous studies, we observed the benefits of normal saline during the 120 min trial, namely MAP rising at T_60 _and CO increasing at T_120_. All 94 patients were treated with crystalloid fluids, but no vasopressors, inotropic agents, colloids or mechanical ventilation were administered within the initial two hours, so the improvement of patient status was certainly caused by normal saline perfusion. This indicates that normal saline can be used for initial volume loading in severe sepsis.

Small volume hypertonic saline is as effective as large-volume isotonic crystalloids in expanding plasma volume and enhancing cardiac output in hemorrhagic shock in animals [42]. Hypertonic saline increases microcirculatory perfusion, presumably by selective arteriolar vasodilatation and by decreasing the swelling of red blood cells and the endothelium [43]. Recent studies demonstrate that hypertonic saline resuscitation reduces the inflammatory reaction in severe sepsis and septic shock [44-47]. In experimental sepsis, the hemodynamic responses to hypertonic saline solution are conflicting: improvement in rats, dogs and pigs [1], no alteration in horses or macaques [13,48], and worsening cardiac contractibility in rabbits [12]. In clinical trials, hypertonic saline with or without a colloidal solution modifies the hemodynamics of patients with sepsis or septic shock. Hypertonic saline/dextran solution improves cardiovascular performance [5], and hypertonic saline/hydroxyethyl starch results in increased cardiac output and pulmonary capillary wedge pressure, but no alteration of O_2 _metabolism [6]. Muller reports that 250 ml 7.5% hypertonic saline without colloid transiently increases the cardiac index and pulmonary capillary wedge pressure in patients with severe sepsis during a 120 min trial [7]. There is a difference in hemodynamic effects between hypertonic saline with colloid and hypertonic saline without colloid. We speculate that the colloid prolongs the effectiveness of the hypertonic solution.

In the present study, 3.5% saline at 5 ml/kg did not alter the CO of patients during the 120 min trial. The effect of 3.5% sodium chloride was very little different from that of 7.5% sodium chloride in Muller's trial. This might be attributable to the difference in osmotic pressure between these two fluids, 1197 mOsm/l in 3.5% sodium chloride and 2565 mOsm/l in 7.5% sodium chloride solution.

Sodium bicarbonate was applied empirically by clinicians before the 1990s in the initial fluid resuscitation of septic shock [15-18]. However, experimental studies of the effect of sodium bicarbonate conflicted. Bollaert et al. demonstrated that sodium bicarbonate treatment does not reduce metabolic cellular injury in skeletal muscle but further reduces MAP in rat models with endotoxic shock [27]. In our study in rabbits [12], 5% sodium bicarbonate induced a decrease in MAP and cardiac contractility, and eventually resulted in the death of the animals. Gossett reports that in ponies with endotoxemia, hypertonic sodium bicarbonate infusion causes blood volume expansion, increases blood bicarbonate concentration and lactate concentration, results in hypokalemia, hypernatremia and hyperosmolality, but does not normalize blood pH [26]. In contrast, our recent study reports that in macaques with early-phase endotoxic shock, normal saline resuscitation further decreases the MAP, cardiac index, left ventricular work index and right ventricular work index, whereas macaques given 5% sodium bicarbonate show moderate increases of cardiac index, left ventricular work index and right ventricular work index. Five percent sodium bicarbonate improves cardiac function in primate models [13]. The above findings indicate that there are differences in the effect of sodium bicarbonate on hemodynamics between primates and non-primates.

The effects of sodium bicarbonate resuscitation in patients with severe sepsis and early-phase septic shock are consistent with those in macaques [13]. In the present study, sodium bicarbonate improved MAP and CO more quickly than normal saline. Sodium bicarbonate manifested a limited beneficial cardiovascular efficiency during the early phase of fluid resuscitation in the patients. Comparing the data from the Hs group with those from the Sb group, a beneficial effect of 5% sodium bicarbonate was that it improved CO and MAP more quickly than 3.5% saline.

In clinical investigations, there are minor differences in the effect of sodium bicarbonate. Mathieu et al. [49] report that administration of sodium bicarbonate does not improve hemodynamic variables in patients with metabolic acidosis, nor does it worsen tissue oxygenation. Cooper [50] found that the MAP responses to sodium bicarbonate and sodium chloride are the same in patients with metabolic acidosis. These data have more than 90% power for detecting a 0.5 l/min difference in mean CO after administration of sodium bicarbonate or sodium chloride. In lactic acidosis accompanied by refractory shock, patients receiving sodium bicarbonate show improvements in neither acid-base balance nor hemodynamics [51]. In the present study, MAP and CO improved more quickly in patients treated with sodium bicarbonate than in patients treated with normal saline or hypertonic sodium chloride. The conflict between our data and those of other authors may be a result of different physiological status before fluid resuscitation: metabolic acidosis in Mathieu's papers, moderate hypotension in the present study and refractory shock in Stacpoole's investigation [51].

Primates, including humans and macaques, appear to tolerate hyperosmotic solutions and acid-alkali balance as potential treatment for septic shock. The severe side-effects of hyperosmotic sodium bicarbonate perfusion in non-primates are hyperosmolarity and hypernatraemia, raised lactic acid and pH, and altered PaCO_2 _and [HCO_3_]^- ^[52-55]. However, in macaques with early-phase endotoxic shock and presenting neither acidosis nor lacticemia, injection of 5 ml/kg 5% sodium bicarbonate does not result in increased osmolarity or plasma sodium, or in altered pH, [HCO_3_]^- ^or lactic acid 60 min after fluid resuscitation [13]. Consistent with the investigation of macaques, patients in the present study with normal pH (pH = 7.38) and receiving sodium bicarbonate showed increased BE, decreased K^+ ^but no alteration of pH, [HCO_3_]^-^, lactic acid, Na^+^, Ca^2+ ^or Cl^- ^during the 120 min trials. The data in macaques and patients indicate that a bolus injection of 5 ml/kg sodium bicarbonate did not affect the acid-basic balance or electrolytes in primates. Sodium bicarbonate might have the same benefits in humans and macaques because primates share similar physiological functions, namely better tolerance to hypertonic solution, volume loading and acid-basic balance [13,14,18]. Therefore, we consider that a bolus injection of 5 ml/kg sodium bicarbonate in the initial fluid resuscitation of severe sepsis is safe – at least it does not worsen the physiological signs of the patients – and this treatment can still be used in cases of severe sepsis and early phase septic shock in humans, but should be deployed with care.

The benefits of the three crystalloid solutions were similar during the 120 min trial and the 8 h follow-up. The MAP and CO during the initial 120 min did not differ among the three groups, and their MAPs had recovered at T_8h _(>70 mmHg). Also, there was no difference among the three groups in observed mortality rates. In addition, fluid resuscitation, whether with normal saline, 3.5% sodium chloride or 5% sodium bicarbonate, did not alter the heart or respiratory rate in any group during the subsequent 120 min. We consider that all the three fluids may be used for initial volume loading in severe sepsis and early-phase septic shock.

Assessments of the severity of organ dysfunction and of physiological status are critical tools for conducting clinical trials, especially sepsis trials. The APACHEA II score, SAPS II score, SOFA and the expected mortality rates were used to judge the severity of patients in previous studies [56]. In the present study, there were no differences in APACHEA II score, SAPS II score, SOFA or the expected mortality rates for APACHE II or SAPS II scores on admission among the three groups, but the age of the Sb group was lower than that of the Ns and Hs groups. Thus, similar pathophysiological scores appeared in the three groups on admission despite this age difference. In general, the populations of the three groups were balanced.

Clinical trials emphasize ethical principles. To ensure a favorable risk-benefit profile, all treatment regimens must provide efficacy and limited risk, with minimal or no emergence of organ injury. In the present study, a regimen was utilized that included a bolus of 5 ml/kg of various solutions at T_0_, no vasoactive agents or colloid before T_120_, and permitted use of vasoactive agents or colloid for hemodynamic stability after T_120_. This design was chosen for the following reasons. First, crystalloid solutions are effective as immediate perfusions in initial volume replacement, but these effects have a short (2–3 h) period of action [34]. In macaque models, the experiments lasted for 60 min, during which time sodium bicarbonate improved myocardial performance and hemodynamics [13]. In the paper by Chrusch and coworkers on the organ metabolism of lactate in dogs with sepsis, the experiment was limited to less than 75 min following fluid resuscitation [57]. Therefore, 120 min duration is suitable for the study of resuscitation by crystalloid solutions. Second, in order to investigate the effect of crystalloid solutions on hemodynamics, vasoactive agents or colloid must not be administered concurrently with this solution resuscitation because vasoactive agents may influence cardiovascular function intensively. But in clinical practice, if fluid resuscitation does not raise blood pressure and restore hemodynamic stability, vasoactive agents should be used to improve the patient's physiological status [39]. Finally, only patients with early-phase septic shock were included in the investigation. The trial included patients with early-phase septic shock because they were tolerant of treatment with single crystalloid solutions within 120 min. The trial excluded patients with final-phase septic shock, because they might require vasoactive agents, colloid or other treatment within 2 h of initial intervention in order to survive; these patients were therefore not suitable for observation.

There are limitations to this study.

First, although the patients were randomly assigned to three groups, the groups were highly heterogeneous in e.g. age and lactic acid level [see Tables 1 &2]. The imbalance between groups could not be avoided in a trial that was conducted over a long period (52 months from Jun 01, 2001 to Oct 31, 2005). Fortunately, there were no significant differences in severity scores between the three groups. Those data indicated that the population was balanced between the three groups. Second, we did not use a pulmonary artery catheter to measure cardiac output, mean central venous pressure or pulmonary arterial wedge pressure, because pulmonary artery catheterization, an invasive technique, is not commonly accepted by patients with early phase septic shock. Therefore, in the present protocol, CO was measured by color Doppler echocardiography. Unfortunately, this technique only analyzed CO; it did not yield essential information about preload assessment (i.e. pulmonary arterial wedge pressure, ventricular working index), oxygen consumption or oxygen delivery. Finally, many leukemic cases were enrolled because those patients were prone to infection, and hypotension was easily detected in inpatients.

## Conclusion

In summary, we demonstrated that all three crystalloid solutions – normal saline, 3.5% hypertonic sodium chloride and 5% sodium bicarbonate – may be used for initial volume loading in patients with severe sepsis and hypotension. Sodium bicarbonate improved MAP and CO earlier than normal saline or hypertonic sodium chloride, which indicates that sodium bicarbonate has a limited benefit for treating severe sepsis in humans.

## Competing interests

The author(s) declare that they have no competing interests.

## Authors' contributions

ZXF participated in the design and management of the investigation. YFL conducted the study and performed the clinical trial at the First People's Hospital of Huai'an City; XQZ conducted the study and performed the clinical trial at the People's hospital of Gaochun County; ZZ conducted the study and performed the clinical trial in the emergency department of the First Hospital of Nanjing; and JSZ conducted the study and performed the clinical trial in the emergency department of the Jiangsu Province Hospital. HMX, WPS and LS performed the statistical analysis and literature search. GPX analyzed the Doppler echocardiography. GQY presented the original idea for the article, and planned and wrote the paper. All authors read and approved the final manuscript.

## Pre-publication history

The pre-publication history for this paper can be accessed here:


